# Collaborative electronic infrastructures to accelerate taxonomic research

**DOI:** 10.3897/zookeys.150.2458

**Published:** 2011-11-28

**Authors:** Vincent S. Smith, Lyubomir Penev

**Affiliations:** 1 Natural History Museum, Cromwell Road, London, SW7 5BD, U.K.; 2 Pensoft Publishers, Sofia, Bulgaria

The discipline of taxonomy lives in a state of perpetual beta, constantly evolving as species hypotheses change to reflect the latest character evidence. Likewise, the electronic infrastructures that underpin taxonomic research are evolving to reflect the latest technical advances. This collection of articles illustrates how advances to research infrastructures are reciprocally changing the practice of taxonomy. The infrastructures have, in part, been developed through the EU funded ViBRANT project. This is the latest in a series of EU funded initiatives that aim to move taxonomy towards a more collaborative, electronic framework in an effort to accelerate the pace of biodiversity research. Through a platform of web-based informatics tools and services, ViBRANT project partners are building a framework that allows distributed groups of scientists to create their own virtual research communities that support biodiversity science. To mark the first year of the ViBRANT project we have brought together a set of reviews, essays and research articles that reflect some of the project highlights and illustrate a number of associated activities. Fittingly, many of the contributions are from researchers who have no direct support from the ViBRANT project, but have used or reviewed some aspect of the infrastructure. This marks an important transition from many EU funded infrastructure projects have typically focused on technical developments, and less on the communities that use these systems.

A detailed review of data issues in the life sciences (Thessen and Patterson 2011) sets the tone for subsequent articles in this special issue, whose contributions broadly fall into three categories. The initial articles consider some of the major infrastructure platforms that support the production and management of biodiversity data. These include the EDIT Platform for Cybertaxonomy, Wiki-based approaches including BioWikiFarm and the Scratchpads Virtual Research Environment. Later articles provide deeper coverage of specialist areas of interest to taxonomic and biodiversity researchers. The topics covered include the mark-up (Penev et al. 2011) and management (King et al. 2011) of taxonomic literature, geospatial assessment of species distributions (Bachman et al. 2011) and licensing issues specific to life science data (Hagedorn et al. 2011). Finally, the special issue closes with a series of research and review papers that provide detailed use cases illustrating how these research infrastructures are being put into practice. These articles make up the majority of this special issue and are subdivided into the sociological analysis of how people are using these infrastructures, as well as the practical experience of biodiversity researchers developing taxonomic data with these systems. Highlights from this section include citizen science approaches to collecting species information by the COMBER Marine observation network (Arvanitidis et al. 2011) and the Australian Bush Blitz programme (Lambkin and Bartlett 2011); use of new tools for data publishing like the Global Biodiversity Information Facility (GBIF) Integrated Publishing Toolkit (IPT) and the DRYAD Data Repository; new forms of publication via “data papers” that allow checklists and identification keys to be formally published as structured datasets (e.g., Narwade et al. 2011); and finally new taxonomic revisions and species descriptions constructed from within the collaborative systems like XPER^2^ and Scratchpads.

This diverse collection of articles illustrates how the paradigm of scholarly communication in taxonomy is being changed by new electronic infrastructures. These support new ways to collaborate and disseminate taxonomic information, facilitating greater reuse of the underlying data. The infrastructures described here are in many cases experimental, but illustrate a number of possible trajectories for how taxonomic data might be assembled and disseminated in the future. These infrastructures will continue to change and evolve, but it now seems certain that the future of taxonomy is increasingly digital, to the point that non-digital work is becoming invisible and perhaps irreverent to the next generation of scholars.
